# Meat–Carbohydrate Dietary Pattern and Elevated Serum Uric Acid in Children and Adolescents: Mediating Role of Obesity in a Cross-Sectional Study

**DOI:** 10.3390/nu17132090

**Published:** 2025-06-24

**Authors:** Guixian Tao, Chunzi Zeng, Jiayi Wan, Wanzhen Zhong, Zheng Su, Shiyun Luo, Jie Huang, Weiwei Zhang, Jun Yuan, Jinxin Zhang, Jichuan Shen, Yan Li

**Affiliations:** 1 School of Public Health, Sun Yat-sen University, Guangzhou 510080, China; taogx@mail2.sysu.edu.cn (G.T.); wanjy26@mail2.sysu.edu.cn (J.W.); zhongwzh6@mail2.sysu.edu.cn (W.Z.); zhjinx@mail.sysu.edu.cn (J.Z.); 2 Guangzhou Center for Disease Control and Prevention, Guangzhou Health Supervision Institute, Guangzhou 510440, China; gzcdc_zengcz@gz.gov.cn (C.Z.); sue1995@smu.edu.cn (Z.S.); luoshy25@mail3.sysu.edu.cn (S.L.); huangjie1026@126.com (J.H.); gzcdczhangww@foxmail.com (W.Z.); yuanjuncom@163.com (J.Y.); 3 School of Public Health, Southern Medical University, Guangzhou 510515, China

**Keywords:** dietary pattern, serum uric acid, obesity, children, adolescents

## Abstract

**Background:** Elevated serum uric acid (SUA) levels in young people have become a significant public health concern. Dietary habits are a key factor influencing SUA levels. This study aimed to investigate dietary patterns (DPs) of children and adolescents and their associations with SUA. **Methods:** This cross-sectional study included children and adolescents in Guangzhou, China. We used structured questionnaires to collect data on demographics, lifestyle, and dietary intake, and we collected blood samples for biochemical analysis. DPs were identified by factor analysis. We used robust linear regression to examine the association between these patterns and SUA levels. Parallel mediation analysis was utilized to assess the mediating role of body mass index (BMI) *Z*-score and waist circumference (WC). **Results:** The study encompassed 4100 children and adolescents between ages 9–17. The median SUA level was 374 (IQR: 319, 438) μmol/L and the prevalence of hyperuricemia was 41.7%. We identified four DPs, including plant-based, snack–beverage, highprotein, and meat–carbohydrate patterns. There was a positive correlation between the meat–carbohydrate pattern and SUA (β = 3.67 μmol/L, 95% CI: 1.22–6.12). The Q4 group of the highprotein pattern was associated with higher SUA levels (9.17 μmol/L, 95% CI: 2.41–15.93) compared to the Q1 group. BMI *Z*-score and WC mediated the association between the meat–carbohydrate pattern and SUA. **Conclusions:** Our findings suggest that BMI *Z*-score and WC mediated the association between the meat–carbohydrate pattern and SUA. This study emphasizes the significance of targeted dietary interventions for weight control in addressing the increasing SUA levels in children and adolescents. Future research could focus on exploring the molecular mechanisms, developing personalized dietary intervention programs, and conducting multicenter prospective cohort studies.

## 1. Introduction

Serum uric acid (SUA) is the end product of the breakdown of purine nucleotides. The sources of SUA can be categorized as exogenous (approximately 20% from dietary purines) and endogenous (approximately 80% synthesized or metabolized by the body), and its excretion relies on both renal and intestinal pathways [1]. When purine is hypermetabolized or its excretion is dysfunctional, SUA accumulation can trigger hyperuricemia, a process that is closely associated with multi-systemic pathologies such as gout [2], cardiovascular disease [3], kidney disease [4], nonalcoholic fatty liver disease [5], and dementia [6]. Notably, elevated SUA in children and adolescents often serves as an early indicator of metabolic disorders. Recent research has shown that the pathological significance of SUA is unique in the child–adolescent population, in which high SUA levels in childhood are significantly associated with metabolic syndrome components such as insulin resistance, obesity, and hypertension [7,8,9]. Thus, maintaining physiological SUA homeostasis is a critical strategy to prevent early metabolic disorders in young people. A study in Chinese youth revealed a rising hyperuricemia prevalence from 16.7% (2009–2015) to 24.8% (2016–2019) [10]. Among US youth, using a uniform cutoff (>5.5 mg/dL), the HUA prevalence was 32.78% and the mean SUA levels were 5.0  ±  1.2 mg/dL [11]. A Brazilian cohort study using the upper-tertile SUA cutoff found that HUA prevalence reached 31.7% in boys (mean SUA 4.35 ± 1.44 mg/dL) and 19.9% in girls (mean SUA 4.02 ± 1.82 mg/dL) [12]. In European children and adolescents, when applying sex-specific thresholds (boys > 420 μmol/L, girls > 370 μmol/L), the HUA prevalence was 12.6% [13]. Therefore, the phenomenon of high SUA levels in children and adolescents underscores its urgency as a public health issue and requires in-depth studies of the impact of modifiable factors on SUA.

Various factors influence SUA levels, including sex, age, medical conditions, obesity, lifestyle factors, and medications [2]. Dietary habits, shaped during childhood and adolescence, are pivotal modifiable determinants of SUA levels. Studies have shown that SUA levels can be reduced by dietary modifications, such as the Dietary Approaches to Stop Hypertension (DASH) diet [14] and the Mediterranean diet [15]. Current dietary guidelines for adults with hyperuricemia and gout recommend limiting the intake of high-purine food and fructose and consuming more fresh vegetables, milk, and dairy products to lower SUA levels [16]. Rapid socio-economic development has transformed the traditional plant-based food pattern of children and adolescents, which is based on grains, tubers, and vegetables, into a pattern high in energy and fat and low in dietary fiber [17]. Meanwhile, rising rates of childhood obesity further exacerbate the negative impact of diet on SUA [18], as obesity exacerbates de novo purine synthesis and insulin resistance [19]. Thus, there is a complex interaction among diet, obesity, and SUA.

Dietary patterns (DPs) consider the complex interrelations among various foods and nutrients as a whole and reflect the actual dietary habits of individuals [20]. In addition, DPs are more consistent over time and have a greater impact on health outcomes than single nutrients or food groups [21]. Therefore, DPs analysis is regarded as a complementary technique to studies on single nutrients or food. Several studies have demonstrated the association among DPs, SUA, and hyperuricemia [22]. However, most evidence is derived from adult populations, neglecting the unique metabolic dynamics of children and adolescents during growth. Furthermore, the mediating effects of general and central adiposity in the DPs–SUA association remain underexplored. The Guangdong region of China has unique dietary habits, such as having a higher intake of aquatic products and consuming rice as the staple food. However, there has been limited research reporting on the effects of these DPs on SUA levels in children and adolescents. Therefore, this study aims to investigate the association between DPs and SUA among children and adolescents in Guangzhou (the capital of Guangdong Province, China) and the mediating role of obesity.

## 2. Materials and Methods

### 2.1. Participants and Study Design

We used data from the Nutrition and Health Surveillance for Students in Guangzhou for this study. This cross-sectional study was carried out from March 2023 to May 2024. We selected five elementary schools (grades 4–6, ages 9–13), five junior high schools (grades 1–2, ages 12–16), and five senior high schools (grade 1, ages 14–17) in Guangzhou. We used random cluster sampling to select two to three classes of students in each of the specified grades. The inclusion criteria were the following: (1) elementary school, junior high school, and senior high school students in the sampled schools, grades, and classes in Guangzhou; (2) signing the informed consent form. The exclusion criteria were the following: (1) those with severe intellectual disabilities or psychiatric problems who were unable to complete the survey; (2) non-school students who were on leave or suspended from school. We implemented the surveys using standardized questionnaires to collect information on their general demographic characteristics, lifestyles, and dietary intake. Parents or guardians completed household questionnaires, while trained investigators conducted school-based surveys, including lifestyle and dietary surveys through face-to-face interviews. All investigators underwent systematic training and passed qualification assessments before data collection. Additionally, participants provided fasting blood samples for biochemical analysis.

**Figure 1:**
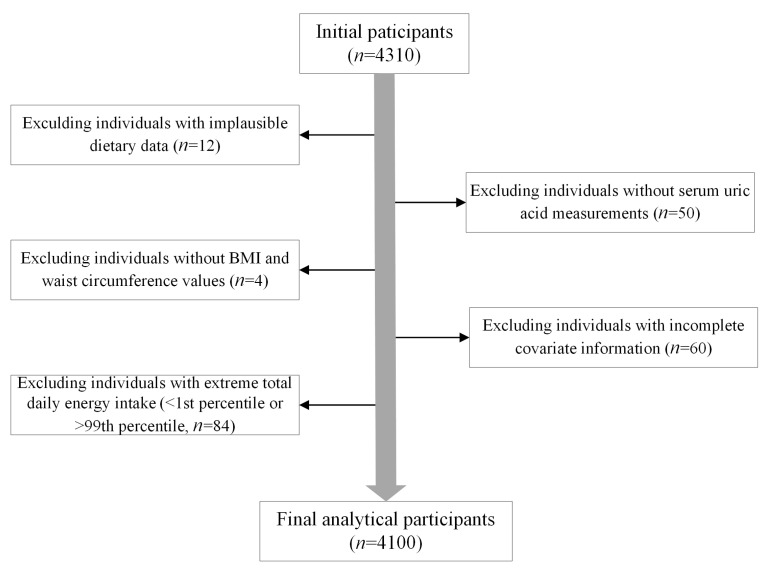
Flowchart of the participant exclusion process.

The flowchart of the participant exclusion process is shown in Figure 1. From the initial participants of 4310 children and adolescents, we excluded 12 individuals with implausible dietary data (defined as missing consumption frequency or portion size data for over 10% of FFQ items), 50 individuals without SUA measurements, 4 individuals without obesity-related anthropometric data, 60 individuals with incomplete covariate information, and 84 individuals exhibiting extreme total daily energy intake (<1st percentile or >99th percentile). After these exclusions, the final analysis of the study included 4100 participants.

### 2.2. Dietary Assessment

The assessment of dietary intake used was a semi-quantitative food frequency questionnaire (FFQ), which collected average habitual dietary intake during the previous month. To ensure estimation accuracy, we used standardized models and colorful photographs of foods to assist participants to recognize food and quantify portion sizes. The FFQ was adapted from the 2015 China National Chronic Non-Communicable Disease and Nutrition Surveillance instrument [23], with modifications made by a multidisciplinary expert panel to reflect the regional dietary habits of Guangzhou’s children and adolescents. We classified food items into 16 categories according to the Chinese Food Composition Table [24]: grains and tubers, beans and bean products, fresh vegetables, pickled vegetables, fungi and algae, fresh fruits, dairy, livestock-derived meat, poultry, animal organs, processed meats, fish and seafood, eggs, nuts, snacks, and beverages (detailed in Table S1). Participants reported consumption frequencies (daily/weekly/monthly) and portion sizes (grams/milliliters) for each item. Total daily intake was calculated by aggregating reported frequencies and standardized portion weights over the 30-day recall period. The daily energy intake was calculated based on the China Food Composition Table. Because of the limitations in FFQ measurement protocols, edible oils and condiments were left out of the analysis.

### 2.3. Physical Examination and Laboratory Tests

We collected height, weight, waist circumference (WC), and blood pressure information. Blood samples were collected from all participants following an overnight fast to measure fasting blood glucose (FBG), triglycerides (TGs), total cholesterol (TC), high-density lipoprotein cholesterol (HDL-C), low-density lipoprotein cholesterol (LDL-C), and SUA. Fasting venous blood (8 mL) was collected using serum separation tubes, and SUA levels were measured by the uricase colorimetric method on an automated biochemistry analyzer (LABOSPECT 008 AS, Hitachi High-Tech Corporation, Tokyo, Japan). This standardized protocol ensured measurement consistency across all samples. Based on the commonly used diagnostic criteria [25,26], hyperuricemia was considered when SUA concentrations exceeded 420 μmol/L in boys and 360 μmol/L in girls in our study.

BMI was calculated by dividing weight (kg) by height squared (m^2^). Based on the World Health Organization (WHO) Growth Reference for children and adolescents aged 5–19 years [27], BMI *Z*-score was computed using the WHO AnthroPlus software version 1.0.4 to ensure alignment with global growth standards. We used sex- and age-specific BMI cutoff values to classify underweight, overweight, and obesity [28,29]. High blood pressure was defined as systolic and/or diastolic blood pressure exceeding the 95th percentile for the same sex, age, and height percentiles [30]. Dyslipidemia was identified as plasma TC ≥ 5.17 mmol/L, LDL-C ≥ 3.36 mmol/L, TGs ≥ 1.46 mmol/L, or HDL-C < 1.03 mmol/L [31]. Impaired fasting glucose was categorized as FBG falling within the range of 5.6–6.9 mmol/L. Diabetes was identified as FBG ≥ 7.0 mmol/L [32].

### 2.4. Assessment of Covariates

We constructed a directed acyclic graph (DAG) in order to use the minimal sufficient adjustment sets in the regression model. Based on the predefined DAG (Figure S1), we determined the following covariates: age, sex, boarding status, physical activity, and sleep duration. The status of boarding was classified as yes or no. Based on the Physical Activity Guidelines for Chinese People (2021) and relevant literature [33,34], physical activity was considered inadequate if it did not meet the recommended levels of moderate–vigorous– intensity physical activity (MVPA) < 1 h/day and outdoor activity < 2 h/day. MVPA is defined as physical activity that results in shortness of breath or an accelerated heart rate, including activities like running, basketball, swimming, or lifting heavy weights. Sleep duration, calculated as the average daily total sleep time (including nighttime and daytime naps), was considered inadequate if reaching <10 h for elementary students, <9 h for junior high students, and <8 h for senior high students [35].

### 2.5. Statistical Analysis

#### 2.5.1. Description and Analysis of the Baseline Characteristics

We summarized and compared the baseline characteristics of participants according to SUA levels. Since SUA was nonnormally distributed, we represented descriptive data for baseline characteristics by median (interquartile range, IQR) for SUA and frequencies (percentages) for categorical variables. Intergroup differences in the distribution of SUA were evaluated using Mann–Whitney U tests or Kruskal–Wallis tests. For post hoc pairwise comparisons following significant Kruskal–Wallis tests, Dunnett’s test was employed, with Bonferroni correction for multiple testing.

#### 2.5.2. Identification of Dietary Patterns

We identified DPs through factor analysis of 16 predefined food groups. We first implemented the Kaiser–Meyer–Olkin test and Bartlett’s sphericity test to assess sampling adequacy and data suitability for factor analysis. Then, we employed a varimax rotation to achieve an orthogonal factor structure and maximize interpretability. The optimal number of factors was determined by eigenvalues (>1), scree plot inflection points, and substantive interpretation of factor composition. Each DP was named according to the major food groups with the highest absolute factor loadings. In addition, we calculated individual factor scores using regression weighting, with higher scores indicating stronger adherence to the corresponding DP. Finally, participants were categorized into quartiles according to each DP score distribution, with Q1 to Q4 indicating scores from lowest to highest.

#### 2.5.3. Association Between Dietary Patterns and Serum Uric Acid and Hyperuricemia

As there are no recognized and consistent diagnostic criteria for hyperuricemia in minors at present, we used SUA levels as the main outcome indicators. Moreover, we also adopted the commonly used epidemiological diagnostic criteria for hyperuricemia. To assess the associations between DPs and SUA levels, robust linear regression was employed. Given the hyperuricemia prevalence exceeding 10% in this cross-sectional study, continued use of odds ratios (OR*s*) could overestimate associations. In this case, we adopted prevalence ratios (PRs) as the preferred measure of associations. Therefore, robust Poisson regression was performed to evaluate DPs–hyperuricemia associations. For each DP, multivariable regression models estimated β coefficients, PR*s*, and 95% confidence intervals (CIs) per 1 standard-deviation (SD) increase in standardized DP scores. Additionally, we categorized DP scores into quartiles, with the lowest quartile serving as the reference category. The median value within each quartile was treated as a continuous variable to assess linear trends.

We established three models for analysis: model 1 was univariate; model 2 was adjusted for sex and age (continuous variable); and model 3 was further adjusted for boarding status (yes/no), physical activity (adequate/inadequate), and sleep duration (adequate/inadequate).

#### 2.5.4. Parallel Mediating Effects of BMI Z-Score and Ln (WC)

We further examined the DPs that were significantly associated with SUA in prior analysis for mediation effects. Obesity was assessed by BMI Z-score and WC. We employed structural equation modeling (SEM) combined with a bootstrap approach to assess the parallel mediating effects of general adiposity (BMI *Z*-score) and central adiposity (WC) in the DPs–SUA association. To account for heteroscedasticity in SEM, SUA and WC values were log-transformed before analysis. A parallel mediation model was constructed to quantify the mediating effect sizes of BMI *Z*-score and natural log-transformed waist circumference [ln (WC)] on the pathway between DPs and SUA, adjusting for covariates specified in model 3. A bootstrap method using iterations of computed samples (5000) was used to validate the significance of the indirect effects. The proportion of mediation effect was calculated as indirect effect/total effect.

#### 2.5.5. Sensitivity Analysis

To ensure the stability and robustness of our findings, we performed comprehensive sensitivity analysis employing three distinct approaches. Firstly, we reselected covariates using a two-step method. In the initial step, we conducted univariate analysis to identify variables that demonstrated an association with the outcome (*p* < 0.2). These variables were then included in the final multivariate model. This approach allows for a more data-driven selection of covariates, reducing the potential for omitted variable bias and ensuring that our results are not unduly influenced by the subjective choice of covariates. Secondly, we excluded outliers of SUA values, which were defined as values falling outside the range of Q1 − 1.5 IQR to Q3 + 1.5 IQR [36]. This step helps to minimize the potential impact of extreme values on the overall results and verify whether our findings are driven by these atypical observations. Lastly, we conducted separate mediation analysis to evaluate the mediating effects of BMI Z-score and ln (WC) individually. This was performed to assess the independent contribution of each adiposity indicator in the association between DPs and SUA, providing a more nuanced understanding of the mediating role of obesity. All statistical analyses were performed using STATA 18.0 and R 4.3.3. All tests were two-sided, and statistical significance was determined when *p* < 0.05.

## 3. Results

### 3.1. Participant Characteristics

We involved a total of 4100 children and adolescents in this study, including 2217 boys (54.07%) and 1883 girls (45.93%). The age range of the participants was 9–17 years old, with a median age of 13.89 (IQR: 11.74, 15.95) years. The median of SUA levels was 374 (IQR: 319, 438) μmol/L and the prevalence of hyperuricemia was 41.7%. Table 1 shows the SUA levels of the study participants across different demographic and lifestyle characteristics. Those with higher SUA levels were more prone to be boys, older age groups, and boarders, with adequate physical activity. Additionally, these children and adolescents were more likely to have a higher nutritional status, have high blood pressure, and have dyslipidemia (all *p* < 0.05).

### 3.2. Dietary Pattern Derivation

**Figure 2:**
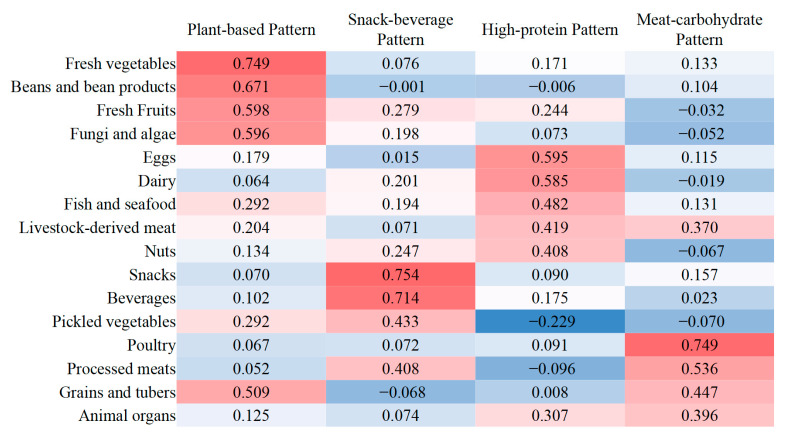
Factor loadings and dietary patterns (DPs) for 16 food groups obtained by factor analysis. The red, white, and blue color spectrum represents the maximum, intermediate, and minimum value of factor loadings, respectively. In each color spectrum, color depth is proportional to the absolute value of the loading.

The Kaiser–Meyer–Olkin index (0.842) and Bartlett’s sphericity test (*p* < 0.001) showed that the correlation among variables was sufficiently strong for factor analysis. We identified four DPs from the dietary information of 4100 participants, accounting for 14.29% (plant-based pattern), 10.71% (snack–beverage pattern), 9.80% (high-protein pattern), and 9.00% (meat–carbohydrate pattern) of total variance, together explaining 43.79% of the variance in 16 food groups intake. The factor-matrix loadings are shown in Figure 2. The plant-based pattern was characterized by high intake of fresh vegetables, beans and bean products, fresh fruits, fungi and algae, and grains and tubers (most were refined grains). The snack–beverage pattern was characterized by high intake of snacks and beverages. The high-protein pattern was characterized by high intake of eggs, dairy, fish and seafood, livestock-derived meat, and nuts. The meat–carbohydrate pattern was characterized by high intake of poultry, processed meats, grains and tubers, animal organs, and livestock-derived meat.

### 3.3. The Associations Among Dietary Patterns and SUA Levels/Hyperuricemia

The result of robust linear regression analysis is presented in Table 2. In model 3, after adjusting for sex, age, boarding status, physical activity, and sleep duration, there was a significantly linear association between the meat–carbohydrate pattern and SUA (*p* = 0.003). Every 1 SD increase in meat–carbohydrate pattern score was associated with 3.67 (95% CI: 1.22–6.12) μmol/L higher in SUA levels. The Q4 group of the meat-carbohydrate pattern was associated with significantly higher SUA levels (8.14 μmol/L, 95% CI: 1.07–15.21, *p* = 0.024) compared to the Q1 group (*p*_trend_ = 0.012). The Q4 group of the high-protein pattern was associated with higher SUA levels (9.17 μmol/L, 95% CI: 2.41–15.93, *p* = 0.008) relative to the Q1 group in model 3 (*p*_trend_= 0.027). There was no significant association among the plant-based pattern, snack–beverage pattern, and SUA (*p* > 0.05).

The result of robust Poisson regression analysis is presented in Table S2. In model 3, after adjusting for covariates, per 1 SD increases in the plant-based pattern (PR = 1.04, 95% CI: 1.01–1.08, *p* = 0.023) and meat-carbohydrate pattern (PR = 1.05, 95% CI: 1.01–1.08, *p* = 0.005) were associated with a higher risk of hyperuricemia. The Q4 group of the high-protein pattern was linked to a higher risk of hyperuricemia (PR = 1.15, 95% CI: 1.04–1.27, *p* = 0.008) compared to the Q1 group (*p*_trend_ = 0.023).

### 3.4. Mediation Analysis

**Figure 3:**
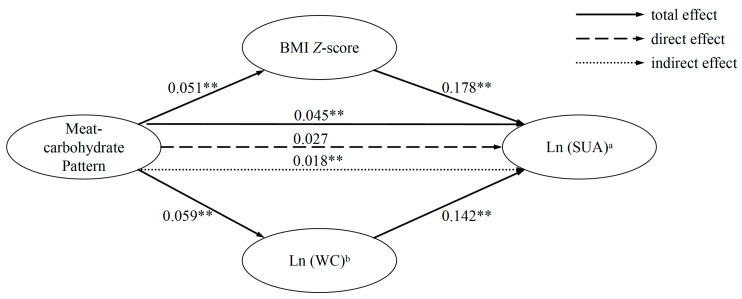
The pathway of parallel mediating effects between meat–carbohydrate pattern and SUA. ^a^ Ln (SUA), log-transformed serum uric acid. ^b^ Ln (WC), log-transformed waist circumference, ** indicates *p* < 0.05.

Considering only the meat–carbohydrate pattern presented a robust significant correlation with SUA in the previous analysis, explorative mediation analysis was conducted to elucidate whether the association between the meat–carbohydrate pattern and SUA was mediated by BMI *Z*-score and ln (WC). As shown in Table 3 and Figure 3, after adjusting for covariates, the indirect effect of BMI *Z*-score was 20.0% (β = 0.009, *p* = 0.007), and the indirect effect of ln (WC) was 17.8% (β = 0.008, *p* = 0.036). The direct effect was of no statistical significance after controlling for mediators (β = 0.027, *p* > 0.05). As a result, the elevation of SUA turned out to be a potential outcome that was mediated by general adiposity and central adiposity.

### 3.5. Sensitivity Analysis of Association Between Dietary Patterns and SUA

Through both univariate screening followed by multivariate modeling and exclusion of participants with extreme SUA levels, we consistently demonstrated robust associations between DPs and SUA (Tables S3 and S4). Furthermore, when separately evaluating the mediating effects of BMI *Z*-score and ln (WC), both adiposity indicators remained significant mediators in meat–carbohydrate pattern–SUA pathways (Table S5).

## 4. Discussion

### 4.1. Summary

In this cross-sectional study among 9–17-year-old children and adolescents in Guangzhou, the median of SUA levels was 374 (IQR: 319, 438) μmol/L, with boys at 412 (IQR: 355, 475) μmol/L and girls at 338 (IQR: 297, 385) μmol/L. The prevalence of hyperuricemia was 41.7%. We identified four distinct DPs through factor analysis: the plant-based pattern, snack–beverage pattern, high-protein pattern, and meat–carbohydrate pattern. Among these patterns, we indicated that the meat–carbohydrate pattern was associated with elevated SUA levels. The higher adherence of the high-protein pattern resulted in significantly higher levels of SUA. The plant-based pattern and snack–beverage pattern were not significantly associated with SUA. Notably, the observed association between the meat–carbohydrate pattern and SUA was mediated by BMI *Z*-score and WC.

### 4.2. Interpretation of SUA Levels

In the study population, boys exhibited higher SUA levels than girls, and these levels rose progressively with age. In fact, the SUA levels increase progressively with age until puberty, with sex differences appearing at approximately 12 years of age, which rises sharply in boys while slightly in girls [37]. This phenomenon has been attributed to the increase in muscle mass stimulated by testosterone during puberty. A study identified that the gender differences in SUA during puberty are associated with higher testosterone in adolescent boys [38], and testosterone can stimulate an increase in muscle mass [39]. Muscle mass serves as the primary reservoir for purines within the body. Moreover, the increase in adenosine triphosphate (ATP) consumption resulting from increased muscle mass induces the release of purine intermediates [40]. Thus, an increase in testosterone is linked to elevated SUA levels and age and gender should be taken into account in the diagnosis of hyperuricemia. To date, there is no international consensus on diagnostic criteria for hyperuricemia in children and adolescents. In our study, the prevalence of hyperuricemia and SUA levels were considerably higher than the national average [41], as well as higher than those reported in Korean youth [8], Japanese children [42], European children and adolescents [13], and US adolescents [11]. This highlights a significant health concern that demands immediate attention and further investigation.

### 4.3. Interpretation of Dietary Patterns

Our study has uncovered four key DPs among children and adolescents: the plant-based pattern, snack–beverage pattern, high-protein pattern, and meat–carbohydrate pattern. These patterns offer a comprehensive lens through which to examine the dietary habits. The plant-based pattern stood out as the most prominent, indicating that plant-based foods still hold a significant place in their diets. The high-protein pattern, characterized by high-quality protein sources such as eggs, dairy, fish, and seafood, is crucial for supporting the growth and development of children and adolescents. However, with improving living standards and the growing availability of snacks and beverages, the snack pattern has emerged as a notable component of their dietary habits. Meanwhile, the meat–carbohydrate pattern, associated with higher calorie and fat intake and lower fruit and vegetable consumption, may pose certain health risks. These findings not only shed light on the current dietary landscape of children and adolescents but also provide a foundation for developing targeted nutritional guidelines to promote healthier dietary habits.

### 4.4. Meat–Carbohydrate Pattern and SUA Levels

SUA levels in children and adolescents are tightly linked to dietary factors. In our study, the meat–carbohydrate pattern was characterized by high intake of poultry, processed meats, grains and tubers, animal organs, and livestock-derived meat. This pattern represents the nutritional transition from food scarcity to diets high in fats and refined carbohydrates that has occurred in China, as evidenced by an excessively higher average daily intake of grains, meat, and poultry compared to the recommendations of the Food Guide Pagoda [43]. Our findings revealed that the meat–carbohydrate pattern positively correlated with SUA. This result highly aligned with the study in Shenzhen, a neighboring city, in which greater adherence to a meat-based diet, including red meats, poultry, and refined grains, showed a positive association with SUA levels in children and adolescents [44]. What is more, a US cohort study revealed that the Western diet was associated with a higher risk of gout [45], which aligned with our study. This association may be ascribed to several factors: (1) Animal-derived food is the main source of dietary purine intake, and high intake and accumulation of purines are associated with elevated SUA [46]; (2) animal food and refined grains are often rich in proinflammatory nutrients. One study has shown that pro-inflammatory diets were associated with a higher risk of hyperuricemia by triggering inflammatory response through complex mechanisms [47]. However, mediation analysis in our study showed a *p*-value = 0.057 for the direct effect. The results were uncertain and needed to be validated by further expansion of the sample size; (3) the high energy content of animal food and refined grains contributes to the elevated risk of overweight and obesity, which are strongly associated with SUA [48,49].

### 4.5. The Mediating Effects of BMI Z-Score and WC

The mediating effects of BMI *Z*-score and WC in our study could echo the above diet–obesity–SUA interpretation. Our results suggest the potential involvement of obesity in the association between the meat–carbohydrate pattern and SUA. This result is consistent with the study in US adults on the association of nutrient patterns to hyperuricemia, in which the indirect effect of obesity was significant, while the direct effect was small or even none [50]. In addition, by using the method of population attributable fractions (PAFs), in a Mendelian randomized study in a European-ancestry sample, BMI exclusively mediated the effects of the four DPs on hyperuricemia [51]. All these findings support our results that diet explains little of the variation in SUA directly, and the association between diet and SUA is largely mediated by obesity. The association between obesity and SUA can be explained by elevated liver synthesis, insulin resistance, and the endocrine role of adipokines [52,53,54]. Based on the parallel mediation model, both BMI *Z*-score and WC independently mediated the association of the meat–carbohydrate pattern with SUA. This suggests that in the prevention and control of high SUA levels in children and adolescents, either general adiposity (BMI *Z*-score) or central adiposity (WC) needs to be included as a breakthrough point for intervention. In Korean adults, elevated BMI and WC, and general and abdominal obesity, may be important risk factors for hyperuricemia in both sexes [55]. Another study in US youth showed that BMI was identified as a risk factor for HUA while no association was found between dietary nutrients and HUA [56]. What is more, among US adults, BMI and WC could mediate the association among SUA, glucose/insulin homeostasis, and inflammation, suggesting that there are possible bidirectional associations between SUA and obesity [57].

### 4.6. High-Protein Pattern and SUA Levels

When analyzing the association between the high-protein pattern (characterized by the high intake of eggs, dairy, fish and seafood, livestock-derived meat, and nuts) and SUA levels, we found that only the highest-propensity group showed statistical significance, and it may be that the corresponding food intake is relatively low in children and adolescents. This pattern reflects one of the characteristics of the Cantonese dietary pattern: sufficient aquatic products; and meat, poultry, eggs, and milk in moderation [58]. It is reported that higher dietary total protein intake is associated with higher SUA levels, and this association is mainly due to the positive correlation between animal protein intake and SUA levels [59]. Potential mechanisms responsible for this association may be the effect of amino acids on purine synthesis and/or exogenous purines in foods rich in animal protein [60]. However, this does not negate the necessity of protein for the growth and development of children and adolescents. It is suggested that their diets need to be optimized for protein proportion.

### 4.7. Plant-Based Pattern, Snack–Beverage Pattern, and SUA Levels

In our study, we observed no statistically significant association between the plant-based pattern and SUA. However, previous studies have reported that plant-based patterns were associated with a lower risk of hyperuricemia [22]. The inconsistency may be attributed to the fact that the SUA-lowering food was offset by the high purine of plant-based food (e.g., beans, mushrooms). The potential interactions among different food ingredients remain worthy of in-depth study. Moreover, there was no statistically significant association between the snack–beverage pattern and SUA in our study. Two studies in adults of other countries have suggested that consumption of sugar-sweetened beverages and a diet based on snacks and processed food were significantly associated with higher SUA levels [61,62]. However, a study in Chinese children and adolescents found that an ultra-processed diet did not show a significant association with SUA levels [44]. In our study, the snack–beverage pattern had no significant association with SUA, which may have a lot to do with children and adolescents’ dietary habits. For participants who primarily consume snacks and beverages instead of meals, the intake of large amounts of snacks and beverages will reduce the intake of other foods, such as purine-rich foods and refined grains. Therefore, the snack–beverage pattern became non-significant. However, this is just a conjecture, and more research is needed to verify the association between the snack–beverage pattern and SUA.

### 4.8. Strengths and Limitations

We have some strengths in this study. First, this study focuses on the population of children and adolescents, and researchers established quality control measures during the survey. Second, our study used a DAG-informed model for covariate adjustment and employed popularly used methods to address mediating effects. The results of the sensitivity analysis showed consistency, which guaranteed the robustness of the findings. Third, blood uric acid is considered as both continuous variable and categorical variable in order to avoid information loss due to the controversy over the thresholds. However, our study has some limitations. First, we did not include traditional soups in our dietary assessment, which may underestimate the effect of DPs on SUA. Second, self-reported dietary data may introduce recall bias, particularly for portion sizes. Third, the study sample from Guangzhou may not represent all children and adolescents, and caution is required when extrapolating the study results.

## 5. Conclusions

This study established four dietary patterns, among which the meat–carbohydrate pattern revealed a significant positive association with SUA. Mediation analysis indicated that BMI *Z*-score and waist circumference mediated these associations. This study emphasizes the significance of targeted dietary interventions aimed at weight management, such as reducing processed meats intake, increasing whole grains intake to replace refined grains, and optimizing protein intake ratios as strategic approaches to addressing the increasing SUA levels in children and adolescents. Future research could focus on exploring the molecular mechanisms, developing personalized dietary intervention programs, and conducting multicenter prospective cohort studies.

## Figures and Tables

**Table 1:** Distribution of serum uric acid levels across demographic and health factors in children and adolescents (*n* = 4100).

Characteristic	*n* (%)	SUA [Median (IQR)] ^a^	*Z/H* ^b^	*p* ^c^
Sex			26.18	**<0.001**
Boy	2217 (54.07)	412 (355, 475)		
Girl	1883 (45.93)	338 (297, 385)		
Age group (year)			257.61	**<0.001**
9–10	591 (14.41)	329 (287, 383)		
11–13 ^d^	1521 (37.10)	369 (318, 430)		
14–17 ^de^	1988 (48.49)	395 (334, 458)		
Boarding status			14.89	**<0.001**
Yes	2413 (58.85)	393 (335, 455)		
No	1687 (41.15)	351 (301, 407)		
Sleep duration			1.17	0.244
Inadequate	2304 (56.20)	376 (320, 438)		
Adequate	1796 (43.80)	373 (316, 437)		
Physical activity			−3.36	**<0.001**
Inadequate	2362 (57.61)	368 (317, 434)		
Adequate	1738 (42.39)	382 (322, 442)		
Education of mother			5.72	0.057
Junior high school or below	1291 (31.49)	378 (320, 443)		
High school	1186 (28.93)	378 (323, 438)		
College degree or above	1623 (39.58)	369 (315, 434)		
Education of father			0.30	0.863
Junior high school or below	1219 (29.73)	376 (319, 439)		
High school	1232 (30.05)	373 (320, 439)		
College degree or above	1649 (40.22)	374 (318, 437)		
Nutritional status			293.21	**<0.001**
Underweight	424 (10.34)	344 (302, 400)		
Normal weight ^f^	2853 (69.59)	366 (314, 424)		
Overweight ^fg^	493 (12.02)	403 (345, 475)		
Obesity ^fgh^	330 (8.05)	458 (382, 522)		
Blood pressure			94.29	**<0.001**
Normal	3637 (88.71)	369 (316, 431)		
High systolic blood pressure ^i^	304 (7.41)	418 (353, 490)		
High diastolic blood pressure ^i^	96 (2.34)	412 (357, 469)		
Both high ^i^	63 (1.54)	431 (369, 496)		
Blood glucose			3.62	0.164
Normal	3926 (95.76)	375 (319, 438)		
Impaired fasting blood glucose	167 (4.07)	362 (322, 434)		
Diabetes	7 (0.17)	316 (279, 404)		
Blood lipid			3.80	**<0.001**
Normal	3131 (76.37)	372 (317, 432)		
Dyslipidemia	969 (23.63)	382 (325, 463)		

Note: Values were reported as number (%) or median (P_25_, P_75_). ^a^ SUA, serum uric acid. ^b^ *Z* is the Mann–Whitney *U* test statistic and *H* is the Kruskal–Wallis test statistic. ^c^ *p* values were calculated by Mann–Whitney *U* tests or Kruskal–Wallis tests. The bold *p*-value means “<0.05”. ^d^ Compared with the 9–10-year-old group, *p* < 0.001; ^e^ compared with the 11–13-year-old group, *p* < 0.001; ^f^ compared with the underweight group, *p* < 0.001; ^g^ compared with the normal-weight group, *p* < 0.001; ^h^ compared with the overweight group, *p* < 0.001; ^i^ compared with the normal group, *p* < 0.001.

**Table 2:** Association between four major dietary patterns and SUA levels (*n* = 4100).

	Quintile of Dietary Pattern Scores							Per 1 SD ^a^ Increase in Dietary Pattern Score	
	Q2		Q3		Q4		*p*_trend_ ^c^		
	β (95% CI ^b^)	*p* ^c^	β (95% CI ^b^)	*p* ^c^	β (95% CI ^b^)	*p* ^c^		β (95% CI ^b^)	*p* ^c^
Plant-based pattern									
Model 1 ^d^	2.84 (−4.75, 10.43)	0.464	5.51 (−2.10, 13.13)	0.156	7.21 (−0.49, 14.92)	0.067	0.263	1.76 (−0.90, 4.42)	0.195
Model 2 ^e^	3.66 (−3.10, 10.43)	0.289	5.96 (−0.85, 12.77)	0.086	7.788 (0.81, 14.77)	**0.029**	0.059	2.07 (−0.35, 4.48)	0.093
Model 3 ^f^	2.57 (−4.12, 9.27)	0.451	5.25 (−1.49,11.98)	0.127	6.11 (−0.80, 13.02)	0.083	0.098	1.96 (−0.43, 4.35)	0.108
Snack–beverage pattern									
Model 1 ^d^	8.51 (0.87, 16.14)	**0.029**	14.95 (7.31, 22.58)	**<0.001**	17.57 (9.86, 25.27)	**<0.001**	**<0.001**	5.40 (2.73, 8.06)	**<0.001**
Model 2 ^e^	3.57 (−3.24, 10.38)	0.304	4.39 (−2.45, 11.24)	0.208	4.02 (−2.91, 10.96)	0.255	0.349	1.50 (−0.89, 3.88)	0.219
Model 3 ^f^	1.48 (−5.28, 8.23)	0.668	1.68 (−5.13, 8.48)	0.629	0.18 (−6.75, 7.12)	0.958	0.993	0.50 (−1.88, 2.88)	0.679
High-protein pattern									
Model 1 ^d^	15.68 (8.07, 23.28)	**<0.001**	9.33 (1.75, 16.91)	**0.016**	15.71 (8.11, 23.30)	**<0.001**	**0.002**	3.61 (0.95, 6.28)	**0.008**
Model 2 ^e^	10.73 (3.96, 17.50)	**0.002**	2.07 (−4.69, 8.83)	0.548	7.41 (0.61, 14.21)	**0.033**	0.168	0.91 (1.47, 3.29)	0.454
Model 3 ^f^	8.95 (2.24, 15.66)	**0.009**	2.33 (−4.36, 9.02)	0.495	9.17 (2.41, 15.93)	**0.008**	**0.027**	1.95 (−0.43, 4.33)	0.108
Meat–carbohydrate pattern									
Model 1 ^d^	12.67 (4.98, 20.36)	**0.001**	21.33 (13.69, 28.97)	**<0.001**	45.38 (37.77, 52.98)	**<0.001**	**<0.001**	16.40 (13.70, 19.06)	**<0.001**
Model 2 ^e^	−0.42 (−7.31, 6.47)	0.905	−0.21 (−7.15, 6.72)	0.952	10.58 (3.48, 17.67)	**0.003**	**0.002**	4.47 (2.01, 6.94)	**<0.001**
Model 3 ^f^	−1.58 (−8.40, 5.24)	0.650	−2.08 (−8.96, 4.80)	0.554	8.14 (1.07, 15.21)	**0.024**	**0.012**	3.67 (1.22, 6.12)	**0.003**

Note: Q1 as the reference. ^a^ SD, standard deviation. ^b^ CI, confidence interval. ^c^ *p*-values were calculated by robust linear regression analysis. The bold *p*-value means “<0.05”. ^d^ Model 1: the crude model. ^e^ Model 2: adjusted for sex and age. ^f^ Model 3: adjusted for model 2 + boarding status, physical activity, and sleep duration.

**Table 3:** The parallel mediating effects of BMI *Z*-score and ln (WC) between meat-carbohydrate pattern and SUA (*n* = 4100) ^a^.

Effects	β (95% CI ^b^)	SE ^c^	*p* ^d^	Mediation Proportion (%) ^e^
Indirect effect via BMI *Z*-score	0.009 (0.003, 0.017)	0.001	**0.007**	20.0
Indirect effect via ln (WC) ^f^	0.008 (0.003, 0.017)	0.001	**0.036**	17.8
Direct effect	0.027 (–0.002, 0.055)	0.003	0.057	/
Total effect	0.045 (0.014, 0.075)	0.004	**0.004**	/

Note: ^a^ Adjusted for age, sex, boarding status, physical activity, and sleep duration. ^b^ CI, confidence interval. ^c^ SE, standard error. ^d^ *p*-values were calculated by structural equation modeling. The bold *p*-value means “<0.05”. ^e^ Mediation proportion (%), indirect effect/total effect. ^f^ Ln (WC), log-transformed waist circumference.

## Data Availability

The datasets generated and/or analyzed during the current study are not publicly available due to privacy but are available from the corresponding authors on reasonable request.

## Supplementary Materials

The following supporting information can be downloaded at https://www.mdpi.com/article/10.3390/nu17132090/s1: Figure S1: Directed acyclic graph (DAG) of association between DPs and SUA; Table S1: Food groups used in the factor analysis; Table S2: Association between four major DPs and hyperuricemia (*n*= 4100); Table S3: Association between DPs and SUA after reselecting covariates (*n* = 4100); Table S4: Association between DPs and SUA after excluding participants with extreme SUA levels (*n* = 4057); Table S5: The simple mediating effect of BMI *Z*-score and ln (WC) between meat–carbohydrate pattern and SUA (*n* = 4100).

## Abbreviations

The following abbreviations are used in this manuscript:

SUA	Serum uric acid
DP	Dietary pattern
BMI	Body mass index
WC	Waist circumference
DASH	Dietary Approaches to Stop Hypertension
FFQ	Food frequency questionnaire
FBG	Blood glucose
TGs	Triglycerides
TC	Total cholesterol
HDL-C	High-density lipoprotein cholesterol
LDL-C	Low-density lipoprotein cholesterol
WHO	World Health Organization
DAG	Directed acyclic graph
MVPA	Moderate–vigorous intensity physical activity
IQR	Interquartile range
OR	Odds ratio
PR	Prevalence ratio
CI	Confidence interval
SD	Standard deviation
SEM	Structural equation modeling
ATP	Adenosine triphosphate
PAF	Population attributable fraction
