# Supplementary material for: Meat–Carbohydrate Dietary Pattern and Elevated Serum Uric Acid in Children and Adolescents: Mediating Role of Obesity in a Cross-Sectional Study
Source: Nutrients. 2025 Jun 24;17(13):2090. doi: 10.3390/nu17132090 (PMC12251295; doi:10.3390/nu17132090)
Supplement: Supplementary file 1 [file nutrients-17-02090-s001.zip › nutrients-3702220-supplementary.pdf]

Supplementary Materials

Figure S1. Directed Acyclic Graph (DAG) of association between DPs and SUA

Table S1 Food groups used in the factor analysis

Table S2 Association between four major DPs and hyperuricemia (*n*= 4,100)

Table S3 Association between DPs and SUA after reselecting covariates (*n*=4,100)

Table S4 Association between DPs and SUA after excluding participants with extreme SUA levels (*n*=4,057)

Table S5 The simple mediating effect of BMI Z-score, ln (WC) between meat-carbohydrate pattern and SUA (*n*=4,100)

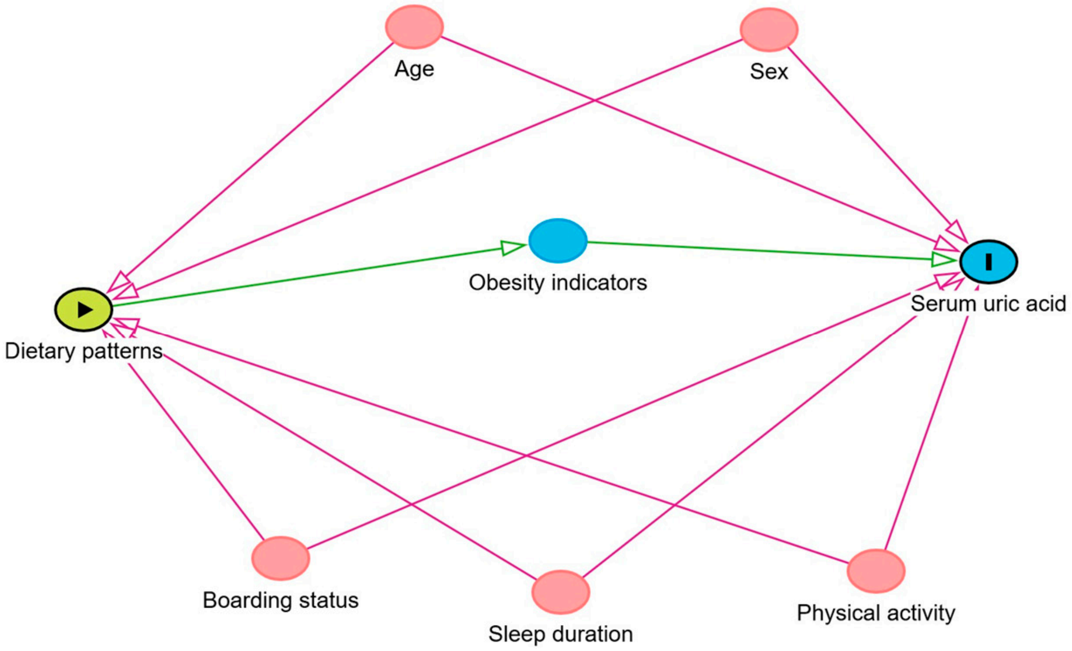

Figure S1 Directed Acyclic Graph (DAG) of association DPs and SUA.

Table S1 Food groups used in the factor analysis.

Food Group	Examples of Food Items
Grains and tubers	Rice and rice products (e.g., steamed rice, rice congee, rice noodles, rice vermicelli, glutinous rice chicken, Zongzi); Wheat and wheat products (e.g., wheat noodles, baozi, jiaozi, steamed bread, youtiao, fried flatbread); Corn and its products (e.g., corn, corn flour, corn flour); Tubers (e.g., potato, sweet potato, Chinese yam, taro)
Beans and bean products	Soybean (e.g., yellow soybean, edamame, black soybean); Soy milk; Tofu; Roll of dried tofu strips; Bean products (e.g., dried tofu, tofu sheets, tofu shreds)
Fresh vegetables	Spinach, lettuce, celery, choy sum, water spinach, coriander, garlic chives
Pickled vegetables	Paocai, suancai
Fungi and algae	Agaricomycetes (e.g., shiitake mushroom, enoki mushroom, oyster mushroom, straw mushroom); Non-mushrooms (e.g., edible tree fungus, white fungus); Kelp; Nori; Seaweed
Fresh fruits	Apple, pear, peach, grape, banana, watermelon, strawberry, orange, blueberry, mango, longan
Dairy	Liquid milk products (e.g., pasteurized milk, pure milk, modified milk); milk powder (e.g., whole milk powder, skimmed milk powder); Yogurt; Dairy products (e.g., milk tablets, cheese, milk lumps, milk tofu)
Livestock-derived meat	Pork, beef, mutton
Poultry	Chicken, duck, goose, pigeon
Animal organs	Bovine offal, porcine offal , and other edible animal by-products (e.g., poultry gizzards, blood curd)
Processed meats	Dry-cured meat, Chinese cured sausage, emulsified ham sausage, canned luncheon meat, processed meatballs, dried pork jerky, dried beef jerky
Fish and seafood	Marine fish (e.g., hairtail, salmon); Freshwater fish (e.g., grass carp, crucian carp); Crustaceans (e.g., shrimp, crabs); Mollusks (e.g., squid, shellfish)
Eggs	Fresh eggs (e.g., chicken eggs, duck eggs, quail eggs); Salted eggs (e.g., salted duck eggs, salted chicken eggs, salted goose eggs); Preserved eggs

Nuts	Sunflower seeds, peanuts, walnuts, cashew nuts, pistachios, hazelnuts, almonds, pine nuts
Snacks	Convenience food (e.g., cookies, cakes, bread, breakfast cereals, pastries, instant noodles, spicy strips); Sugary products (e.g., granulated sugar, candies, chocolate, preserved fruits); Western-style fast food (e.g., hamburgers, fried chicken); Puffed snacks (e.g., potato chips, shrimp crackers)
Beverages	Sugar-free beverages, carbonated beverages, fruit/vegetable juices, plant protein beverages, dairy-containing beverages, tea (and its types) beverages, plant-based beverages, milk tea beverages, sports beverages, coffee (and its types) beverages, ice cream

Table S2 Association between four major DPs and hyperuricemia (*n*= 4,100).

	Quintile of dietary pattern score							Per 1 *SD*[a] increase in dietary pattern score	
	Q2		Q3		Q4		P_{trend}[c]		
	PR (95% *CI*[b])	*P*[c]	*PR* (95% *CI*[b])	*P*[c]	*PR* (95% *CI*[b])	*P*[c]		*PR* (95% *CI*[b])	*P*[c]
**plant-based pattern**									
Model 1[d]	1.00 (0.90, 0.91)	0.932	0.98 (0.88, 1.08)	0.644	1.03 (0.93, 1.14)	0.570	0.813	1.01 (0.97, 1.05)	0.597
Model 2[e]	1.02 (0.92, 1.13)	0.676	1.02 (0.92, 1.13)	0.740	1.11 (1.00, 1.23)	**0.042**	0.051	1.04 (1.01, 1.08)	**0.027**
Model 3[f]	1.01 (0.92, 1.12)	0.782	1.01 (0.91, 1.12)	0.854	1.10 (0.99, 1.22)	0.073	0.062	1.04 (1.01, 1.08)	**0.023**
**snack-beverage pattern**									
Model 1[d]	1.06 (0.96, 1.18)	0.260	1.12 (1.01, 1.24)	**0.032**	1.12 (1.01, 1.24)	**0.037**	**0.032**	1.04 (1.01, 1.08)	**0.013**
Model 2[e]	1.02 (0.92, 1.14)	0.676	1.04 (0.94, 1.15)	0.452	1.03 (0.93, 1.14)	0.584	0.539	1.02 (0.99, 1.06)	0.171
Model 3[f]	0.99 (0.89, 1.10)	0.891	1.00 (0.91, 1.11)	0.964	0.98 (0.88, 1.09)	0.695	0.828	1.01 (0.98, 1.05)	0.510

**high-protein pattern**									
Model 1[d]	1.18 (1.07, 1.31)	**<0.001**	1.03 (0.93, 1.15)	0.533	1.12 (1.01, 1.24)	**0.041**	0.210	1.01 (0.98, 1.05)	0.436
Model 2[e]	1.16 (1.05, 1.29)	**0.004**	1.02 (0.92, 1.14)	0.705	1.12 (1.01, 1.24)	**0.032**	0.126	1.02 (0.98, 1.10)	0.404
Model 3[f]	1.15 (1.03, 1.27)	**0.009**	1.03 (0.93, 1.15)	0.589	1.15 (1.04, 1.27)	**0.008**	**0.023**	1.03 (0.99, 1.06)	0.120
**meat-carbohydrate pattern**									
Model 1[d]	1.01 (0.91, 1.13)	0.829	1.09 (0.98, 1.21)	0.125	1.27 (1.14, 1.40)	**<0.001**	**<0.001**	1.10 (1.07, 1.13)	**<0.001**
Model 2[e]	0.97 (0.87, 1.08)	0.537	0.99 (0.89, 1.10)	0.810	1.10 (0.99, 1.22)	0.083	**0.034**	1.05 (1.02, 1.09)	**0.001**
Model 3[f]	0.95 (0.86, 1.06)	0.397	0.97 (0.87, 1.08)	0.565	1.07 (0.97, 1.19)	0.192	0.077	1.05 (1.01, 1.08)	**0.005**

Note. Q1 as the reference. [a]*SD*, standard deviation. [b]*CI*, confidence interval. [c]*P* values were calculated by robust Poisson regression analysis. Figure in bold indicates $P < 0.05$. [d]Model 1: the crude model. [e]Model 2: adjust for sex and age. [f]Model 3: adjust for model 2 + boarding status, physical activity, and sleep duration.

Table S3 Association between DPs and SUA after reselecting covariates (*n*=4,100)[a].

	Quintile of dietary pattern score							Per 1 *SD*[b] increase in dietary pattern score	
	Q2		Q3		Q4				
	β (95% *CI*[c])	*P*[d]	β (95% *CI*[c])	*P*[d]	β (95% *CI*[c])	*P*[d]	P_{trend}[d]	β (95% *CI*[c])	*P*[d]
**plant-based pattern**	2.14 (–4.28, 8.55)	0.513	5.02 (–1.43, 11.47)	0.127	4.14 (–2.49, 10.76)	0.221	0.304	1.05 (–1.246, 3.34)	0.371
**snack-beverage pattern**	4.47 (–2.01, 10.95)	0.176	4.60 (–1.93, 11.13)	0.167	4.26 (–2.38, 10.90)	0.208	0.309	1.87 (–0.412, 4.14)	0.108
**high-protein pattern**	8.29 (1.88, 14.71)	**0.011**	2.31 (–4.12, 8.74)	0.481	10.39 (3.89, 16.89)	**0.002**	**0.008**	2.26 (–0.031, 4.54)	0.053

**meat-carbohydrate pattern**	−0.72 (−7.25, 5.80)	0.828	−0.52 (−7.10, 6.05)	0.876	6.98 (0.230, 13.73)	**0.043**	**0.028**	3.50 (1.16, 5.83)	**0.003**

Note. Q1 as the reference. [a]Adjust for sex, age, boarding status, physical activity, education of mother, nutritional status, blood pressure, blood lipid, and blood glucose. [b]*SD*, standard deviation. [c]*CI*, confidence interval. [d]*P* values were calculated by robust linear regression analysis. Figure in bold indicates $P < 0.05$.

Table S4 Association between DPs and SUA after excluding participants with extreme SUA levels (*n*=4,057)[a].

	Quintile of dietary pattern score							Per 1 *SD*[b] increase in dietary pattern scores	
	Q2		Q3		Q4		P_{trend}[d]		
	β (95% *CI*[c])	*P*[d]	β (95% *CI*[c])	*P*[d]	β (95% *CI*[c])	*P*[d]		β (95% *CI*[c])	*P*[d]
**plant-based pattern**	2.90 (−3.69, 9.50)	0.388	5.34 (−1.30, 11.98)	0.115	6.53 (−0.30, 13.36)	0.061	0.073	2.01 (−0.28, 4.43)	0.084
**snack-beverage pattern**	0.77 (−5.89, 7.42)	0.821	1.11 (−5.59, 7.80)	0.746	−0.14 (−6.97, 6.69)	0.968	0.937	0.43 (−1.91, 2.77)	0.718
**high-protein pattern**	8.92 (2.32, 15.53)	**0.008**	2.32 (−4.27, 8.91)	0.490	8.48 (1.81, 15.15)	**0.013**	**0.043**	1.88 (−0.46, 4.23)	0.116
**meat-carbohydrate pattern**	−1.75 (−8.47, 4.96)	0.609	−1.99 (−8.75, 4.78)	0.565	7.19 (0.22, 14.15)	**0.043**	**0.022**	3.19 (0.76, 5.62)	**0.010**

Note. Q1 as the reference. [a]Adjust for sex, age, boarding status, physical activity, and sleep duration. [b]*SD*, standard deviation. [c]*CI*, confidence interval. [d]*P* values were calculated by robust linear regression analysis. Figure in bold indicates $P < 0.05$.

Table S5 The simple mediating effect of BMI Z-score, ln (WC) betwee meat-carbohydrate pattern and SUA (*n*=4,100)[a].

Effects	β (95% *CI*[b])	*SE*[c]	*P*[d]	Mediation proportion (%)[e]
**BMI Z-score**				31.8
Total effect	0.044 (0.013, 0.073)	0.004	**0.004**	
Direct effect	0.030 (0.001, 0.058)	0.003	**0.039**	

Indirect effect	0.014 (0.005, 0.024)	0.001	**0.003**	
**Ln (WC)[f]**				38.6
Total effect	0.044 (0.013, 0.073)	0.004	**0.005**	
Direct effect	0.027 (0.001 ,0.058)	0.003	0.060	
Indirect effect	0.017 (0.005, 0.024)	0.001	**0.002**	

Note. [a]Adjusted for age, sex, boarding status, physical activity, and sleep duration. [b]*CI*, confidence interval. [c]*SE*, standard error. [d]*P* values were calculated by structural equation modeling. Figure in bold indicates $P < 0.05$. [e]Mediation proportion (%), indirect effect / total effect. [f]Ln (WC), log-transformed waist circumference.
